# The Tyrosine Phosphatase SHP-1 Regulates Hypoxia Inducible Factor-1α (HIF-1α) Protein Levels in Endothelial Cells under Hypoxia

**DOI:** 10.1371/journal.pone.0121113

**Published:** 2015-03-23

**Authors:** Stefan K. Alig, Yvonn Stampnik, Joachim Pircher, Raffaela Rotter, Erik Gaitzsch, Andrea Ribeiro, Markus Wörnle, Florian Krötz, Hanna Mannell

**Affiliations:** 1 Walter Brendel Centre of Experimental Medicine, University of Munich, Munich, Germany; 2 Department of Internal Medicine III, University of Munich, Munich, Germany; 3 Department of Internal Medicine I, University of Munich, Munich, Germany; 4 Department of Internal Medicine IV, University of Munich, Munich, Germany; 5 Interventional Cardiology, Starnberg Community Hospital, Starnberg, Germany; University of Kentucky, UNITED STATES

## Abstract

**Introduction:**

The tyrosine phosphatase SHP-1 negatively influences endothelial function, such as VEGF signaling and reactive oxygen species (ROS) formation, and has been shown to influence angiogenesis during tissue ischemia. In ischemic tissues, hypoxia induced angiogenesis is crucial for restoring oxygen supply. However, the exact mechanism how SHP-1 affects endothelial function during ischemia or hypoxia remains unclear. We performed *in vitro* endothelial cell culture experiments to characterize the role of SHP-1 during hypoxia.

**Results:**

SHP-1 knock-down by specific antisense oligodesoxynucleotides (AS-Odn) increased cell growth as well as VEGF synthesis and secretion during 24 hours of hypoxia compared to control AS-Odn. This was prevented by HIF-1α inhibition (echinomycin and apigenin). SHP-1 knock-down as well as overexpression of a catalytically inactive SHP-1 (SHP-1 CS) further enhanced HIF-1α protein levels, whereas overexpression of a constitutively active SHP-1 (SHP-1 E74A) resulted in decreased HIF-1α levels during hypoxia, compared to wildtype SHP-1. Proteasome inhibition (MG132) returned HIF-1α levels to control or wildtype levels respectively in these cells. SHP-1 silencing did not alter HIF-1α mRNA levels. Finally, under hypoxic conditions SHP-1 knock-down enhanced intracellular endothelial reactive oxygen species (ROS) formation, as measured by oxidation of H_2_-DCF and DHE fluorescence.

**Conclusions:**

SHP-1 decreases half-life of HIF-1α under hypoxic conditions resulting in decreased cell growth due to diminished VEGF synthesis and secretion. The regulatory effect of SHP-1 on HIF-1α stability may be mediated by inhibition of endothelial ROS formation stabilizing HIF-1α protein. These findings highlight the importance of SHP-1 in hypoxic signaling and its potential as therapeutic target in ischemic diseases.

## Introduction

Src homology region 2 domain-containing phosphatase-1 (SHP-1), a nonreceptor-type protein tyrosine phosphatase (PTP), is mainly expressed in hematopoietic cells and epithelial tissue like endothelial cells [1, 2]. We previously showed the importance of SHP-1 in endothelial function and homeostasis. We found SHP-1 to protect the endothelium from adhesion molecule upregulation and thrombosis under inflammatory conditions *in vitro* and *in vivo* [3]. Furthermore, another study from our group showed that SHP-1 also negatively regulates VEGF mediated endothelial cell signaling, by inhibiting the formation of reactive oxygen species (ROS) [2]. ROS is important for angiogenic processes and conditions where angiogenesis is induced, such as hypoxia or ischemia, are known to increase endothelial ROS formation [4]. Hypoxia dependent vessel sprouting (angiogenesis) is mediated by the transcription factor Hypoxia inducible factor-1 (HIF-1) [4–6], a major regulator of cellular adaption to hypoxia consisting of an oxygen dependently regulated α-subunit and a constitutively expressed β-subunit [7, 8]. Under normoxic conditions the HIF-1α subunit is marked for degradation by prolylhydroxylases and subsequently degraded by the proteasomal degradation system. Under hypoxic conditions however, the degradation is inhibited with accumulation of HIF-1α as a result [9, 10]. ROS have been shown to stabilize HIF-1α during hypoxia by preventing its degradation [11]. During hypoxia HIF-1 functions as a transcription factor for proteins such as the vascular endothelial growth factor (VEGF), which is of crucial importance for hypoxia driven endothelial cell proliferation. It has been shown that HIF-1 mediated VEGF secretion is essential for hypoxia induced endothelial cell proliferation by an autocrine loop mechanism [6, 12]. Interestingly, SHP-1 has been shown to inhibit endothelial cell proliferation and survival under hypoxic conditions [13, 14] and knock-down of SHP-1 results in improved angiogenesis in hindlimb ischemia [14]. Moreover, in vivo studies show the potential of SHP-1 inhibition to decrease necrosis size in myocardial infarction [15, 16] and ischemic stroke [17].

These findings highlight the importance of SHP-1 in hypoxia and ischemic diseases as well as its therapeutic potential. However, the mechanism how SHP-1 mediates these effects in hypoxia/ ischemia is not fully understood. In particular an involvement of SHP-1 in the regulation of HIF-1α has not yet been investigated.

In this study, we show that an inhibition of SHP-1 in human microvascular endothelial cells (HMEC) increases endothelial proliferation and VEGF secretion during hypoxia. Furthermore, we find that these effects were due to enhanced HIF-1α protein levels, possibly by enhanced ROS formation.

## Materials and Methods

### Chemicals

Mouse HIF-1α antibody used for immunoprecipitations was purchased from BD Biosciences (#610959, Heidelberg, Germany) and rabbit HIF-1α antibody used for western blotting was obtained from Merck Millipore (#04-1006,Darmstadt, Germany). Mouse (D-11, #sc-7289) and Rabbit (C-19, #sc-287) SHP-1 for immunoprecipitations and western blotting respectively and mouse VEGF (C-1, #sc-7269) antibodies were from Santa Cruz biotechnology (Heidelberg, Germany). Mouse phospho-tyrosine antibody was from Merck Millipore (# 05-321, Darmstadt, Germany). Beta-Actin antibody was from Cell Singaling Technology (#4970, Danvers, MA, USA). Horseradish peroxidase-conjugated antibodies were from Calbiochem (Darmstadt, Germany) and fluorescently labeled secondary antibodies were from Invitrogen (Darmstadt, Germany). MG132 and Echinomycin were from Merck (Darmstadt, Germany). All other chemicals were purchased from Sigma-Aldrich (Taufkirchen, Germany).

### Cell lines and cell culture

HMEC were provided by Ades et al.[18] and cultured as described previously [19]. HMEC were put on starvation medium (DMEM, 1% fetal calf serum) 24 hours prior to experiments unless stated otherwise.

### Hypoxic environment

Cells were exposed to hypoxia in an appropriate chamber purchased from Cell Systems (Troisdorf, DE), which was flooded with 15–20 l/min of an anoxic gas mixture (5% CO_2_, 95% N_2_) for 4 minutes. The cell medium was bubbled with the same gas mixture for 30 minutes before usage. The hypoxia chamber was placed in an incubator at 37° C for either 4 or 24 hours. O_2_ partial pressure in the cell medium reached an equilibrium after appr. 30–60 minutes at 8±2 mmHg, which equals an O_2_ concentration of 1±0.2%. This hypoxic environment was shown to induce HIF-1α expression after 4 hours, as well as VEGF synthesis and secretion after 24 hours.

### Transfection of antisense oligonucleotides (AS Odn)

HMEC were transfected with specific SHP-1 AS Odn (5‘-ccttgagcagggtctctgcatcc-3‘) or control Odn (5‘-cccttatttactactttcgc-3‘) respectively in a concentration of 70 nM using the magnetofection method [20, 21] and the Effectene kit from Qiagen (Hilden, Germany) as previously described [2]. Odn were synthesized by Eurofins MWG Operon (Ebersberg, Germany). Experiments were performed 24 hours after transfection.

### Plasmid transfer

HMEC were transfected with either SHP-1 wildtype, SHP-1 CS (dominant negative form due to mutation of Cys453 to Ser453 in the catalytic domain) or SHP-1 E74A (constitutively active form due to mutation of the Glu74 to Ala74 in the N-SH2 domain) plasmids using PeqFECT DNA (PEQLAB Biotechnologie Erlangen, Germany) according to the manufacturer’s protocol. Experiments were performed 48 to 72 hours after transfection. Plasmids were obtained from Addgene (www.addgene.org).

### Cell extracts for western blot analysis and immunoblotting

Protein lysates were prepared and protein content quantified as described previously [2]. Lysates were then subjected to western blot analysis as described elsewhere [2].

### Immunofluorescence staining

For immunofluorescent microscopy of SHP-1 and HIF-1α, cells were grown to confluence on 8-well microscope slides from IBIDI (Martinsried, Germany) and stained and visualized as previously described [22]. Quantification of fluorescent intensity was performed by pixel measurements using AxioVision 4.8 from Zeiss (Jena, Germany).

### Immunoprecipitation

Immunoprecipitations were performed using μMACS Protein G MicroBeads and MACS separation columns from Miltenyi Biotec (Bergisch Gladbach, Germany) according to the manufacturer’s protocol. The precipitates were quantified by western blotting.

### Proliferation measurement

To quantify proliferation and cell viability protein concentrations of single wells of a 24-well plate were measured by Biuret method as previously described [2].

### Quantification of VEGF synthesis and secretion

VEGF synthesis was determined by flow cytometry. Cells were exposed to hypoxia/normoxia without prior starvation. Subsequently, cells were detached using Accutase (PAA Laboratories, Pasching, Austria), pelleted, fixated with 5% formaldehyde solution for 10 min, pelleted, permeabilized with 0.1% Triton solution for 5min, rinsed, incubated with VEGF antibody for 1 hour (1:200 in Phosphate buffered saline (Pbs) supplemented with calcium), rinsed and incubated with FITC labeled secondary antibody (1:400 in Pbs supplemented with calcium) for 30 min, pelleted and dissolved in Pbs supplemented with calcium for measurement by flow cytometry using a FACS Canto II (BD Biosciences, Heidelberg, Germany). To control for background staining, control cells were stained with secondary antibody only.

VEGF secretion in the supernatant was measured using Quantikine Human VEGF Immunoassay from R&D Systems (Minneapolis, MN, USA) according to the manufacturer’s protocol.

### RNA isolation and quantitative real time PCR

Total RNA was isolated using the PeqGOLD total RNA kit (Peqlab Erlangen, Germany). A total of 2 μg RNA was transcribed to DNA by using SuperScript Reverse Transcriptase (Life Technologies, DE). Real time PCR was performed on TaqMan ABI 7700 (PE Applied Biosystems, Darmstadt, DE). TaqMan gene expression assays for HIF-1α (#Hs00153153_m1) and GAPDH (#4310884E) were used (Life Technologies, DE).

### Measurement of reactive oxygen species (ROS)

Intracellular ROS formation was analyzed by quantifying the ROS dependent oxidation of H_2_–2’, 7’-Dichlorofluorescein (H_2_-DCF) to fluorescent DCF. Cells were incubated with 20 μM H_2_DCF diacetate during normoxia/hypoxia, detached using Accutase (PAA Laboratories, Pasching, Austria), pelleted, rinsed and dissolved in PBS supplemented with calcium for instant measurement by flow cytometry using a FACS Canto II (BD Biosciences, Heidelberg, Germany). Superoxide production was detected by dihydroethidium (DHE) fluorescence. Cells were incubated with DHE (20 μM) during hypoxia/normoxia followed by detachment using Accutase (PAA Laboratories, Pasching, Austria). Cells were pelleted, rinsed and dissolved in PBS supplemented with calcium for instant measurement by flow cytometry using a FACS Canto II (BD Biosciences, Heidelberg, Germany). Median fluorescent units were used for the analysis.

### Statistical analysis

Data were analyzed using Student's t-test, Wilcoxon rank sum test or one-way ANOVA as appropriate. All data are presented as means ± SEM. Results were considered significant at an error probability level of P < 0.05.

## Results

### Expression and localization of SHP-1 and HIF-1α in HMEC

**Fig 1:**
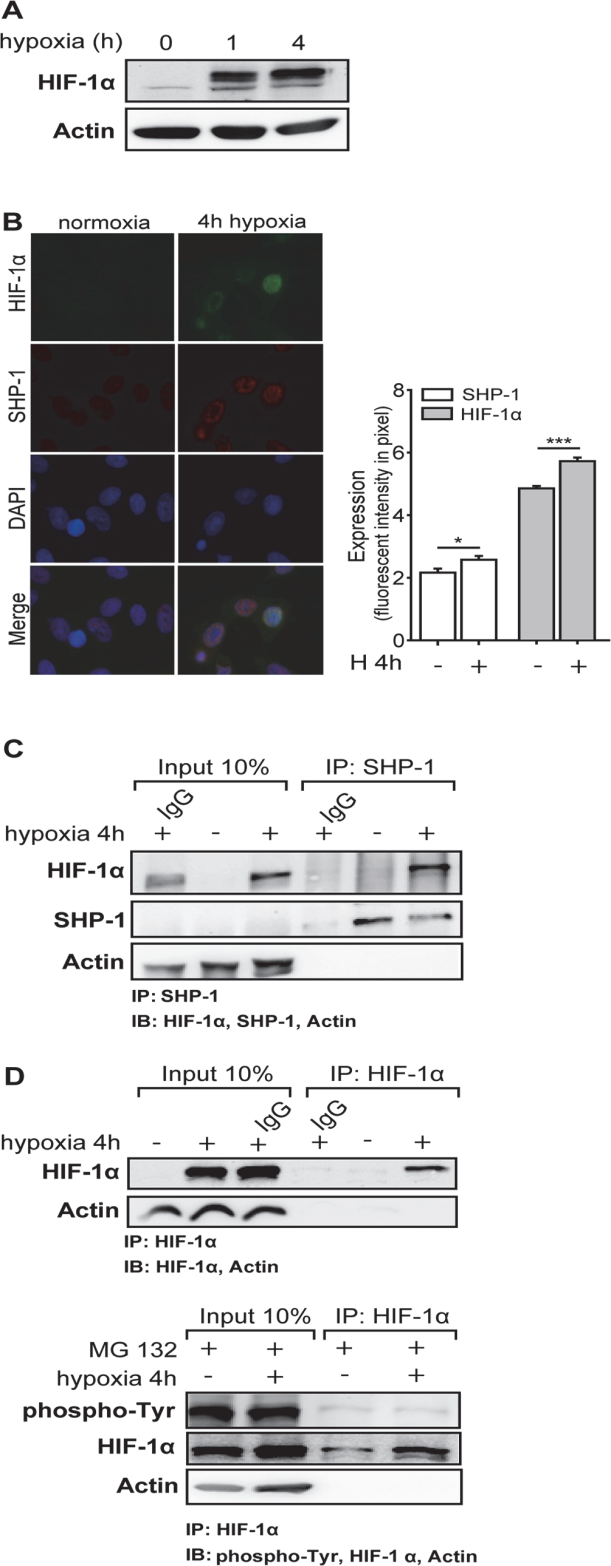
SHP-1 expression and localization during hypoxia. (A) The induction of HIF-1α protein levels by 1h and 4h hypoxia was confirmed by western blot (n = 4). (B) HIF-1α (green) and SHP-1 (red) were detected by immunofluorescence staining. DAPI (blue) was used to visualize nuclei. HIF-1α was induced by hypoxia (***p<0.001; n = 6). SHP-1 expression was also slightly induced by hypoxia (p<0.05; n = 6). Both SHP-1 and HIF-1α were shown to be predominantly located in the nucleus with moderate expression in the cytoplasm. Graphs next to photos show fluorescent intensities of SHP-1 and HIF-1α (n = 6 of 6 visual fields/sample) (C) Immunoprecipitation was performed for SHP-1. HIF-1α could be detected in precipitates of SHP-1 (n = 6). IgG: IgG isotype control antibody. (D) HIF-1α could successfully be immunoprecipitated by a specific antibody (lane 6 upper blot) and is tyrosine phosphorylated under normoxia and hypoxia as seen in MG132 (10μM) treated cells (lane 3 and 4 lower blot, n = 3). IgG: IgG isotype control antibody.

First, we analyzed expression and localization of SHP-1 and HIF-1α in HMEC during normoxia and hypoxia. HIF-1α protein was induced by 1h and 4h hypoxia (western blot: n = 4, Fig. 1A). HIF-1α induction by 4h hypoxia could be confirmed by immunofluorescent staining (p<0.001; immunofluorescence: n = 6, Fig. 1B). On the contrary, SHP-1 protein was expressed during normoxic as well as hypoxic conditions but showed a slight increase in expression upon hypoxia (immunofluorescence: p<0.05; n = 6, Fig. 1B). Furthermore, analysis revealed that both proteins are predominantly located in the nucleus of HMEC with only moderate expression in the cytoplasm (n = 6, Fig. 1B). Additionally, immunoprecipitation of SHP-1 was performed and HIF-1α was detected in the precipitate (n = 6, Fig. 1C). SHP-1 protein could only be detected after performing immune precipitation with an antibody recognizing SHP-1 and not in 30μg of whole cell lysates indicating a relatively low expression level in endothelial cells. To investigate if HIF-1α is tyrosine phosphorylated in endothelial cells, immunoprecipitations of HIF-1α was performed followed by detection of phospho-tyrosine residues by western blot. As seen in Fig. 1D (upper blot lane 4), no HIF-1α was precipitated when using an IgG isotype control antibody and HIF-1α could successfully be precipitated when using a specific antibody (upper blot lane 6, n = 3). To analyze possible differences in HIF-1α tyrosine phosphorylation in normoxia and hypoxia, the immunoprecipitation was performed again in cells treated with the proteasome inhibitor MG132 to achieve equal HIF-1α amounts during both conditions. As seen in Fig. 1D (lower blot) HIF-1α is tyrosine phosphorylated but no difference between normoxia and hypoxia could be detected (n = 3).

### SHP-1 regulates endothelial cell growth and VEGF synthesis during hypoxia

To investigate the role of SHP-1 on endothelial cell growth under hypoxic conditions SHP-1 was knocked-down by transfecting HMEC with specific antisense oligodesoxynucleotides (AS-Odn). The efficacy of this technique in endothelial cells has been described earlier by our group [2].

**Fig 2:**
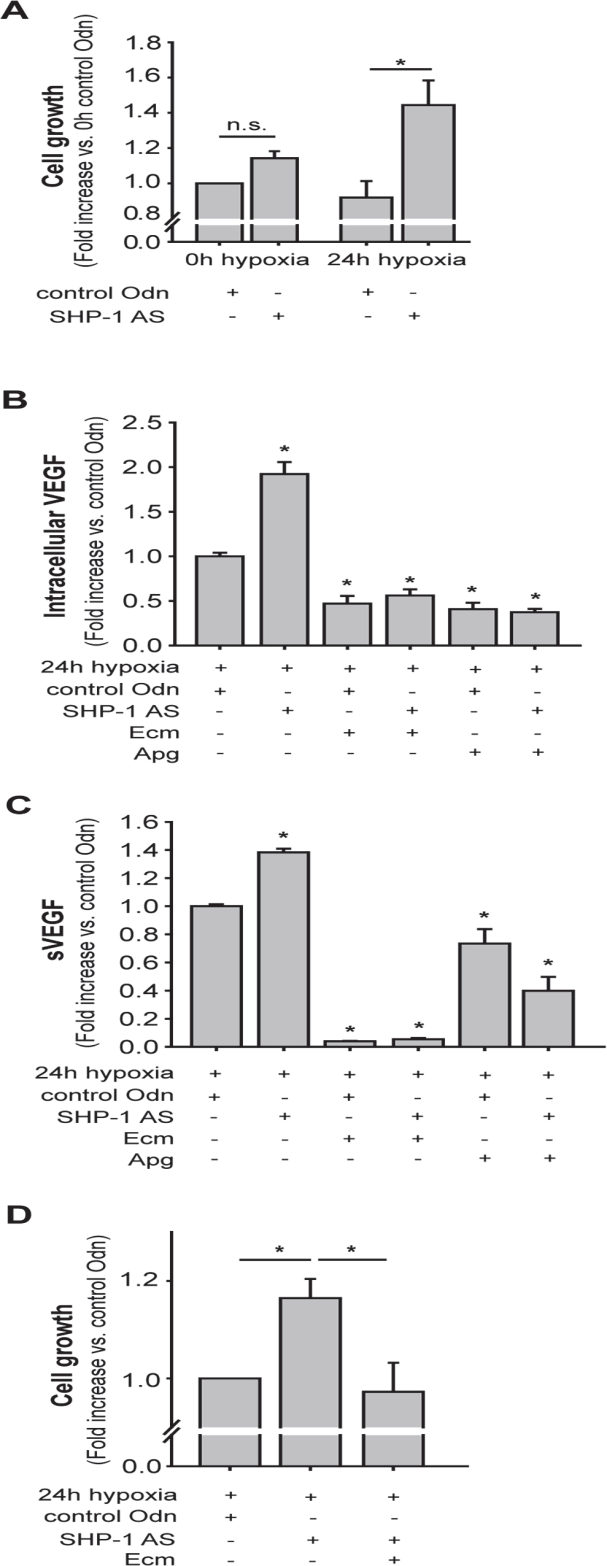
SHP-1 knock-down increased cell growth and VEGF synthesis and secretion during hypoxia. (A) Cell growth was quantified by measurement of total protein content of single wells. Knock-down of SHP-1 resulted in increased cell growth during 24 hours of hypoxia compared to control Odn (*p<0.05, n = 18). (B) Intracellular VEGF was measured by FACS. SHP-1 knock-down led to increased levels of intracellular VEGF after 24 hours of hypoxia (*p<0.05, n = 10), which was blocked by echinomycin (Ecm, 10ng/ml) (*p<0.05 vs. control Odn; n = 10) as well as apigenin (Apg, 50μM) (*p<0.05 vs. control Odn; n = 10). (C) SHP-1 knock-down resulted in elevated VEGF concentration in supernatants of hypoxic HMECs, as measured by ELISA (*p<0.01, n = 18). Inhibition of HIF-1α activity by echinomycin (10ng/ml) or apigenin (50μM) reduced this in both control Odn and SHP-1 AS-Odn treated cells (*p<0.05 vs. control Odn; n = 8). (D) The increased cell growth during hypoxia by SHP-1 knock-down could be prevented by adding the HIF-1 inhibitor echinomycin (Ecm, 10 ng/ml) (*p<0.05, n = 11).

Silencing of SHP-1 led to increased cell growth during 24 hours of hypoxia (p<0.05; n = 18, Fig. 2A) compared to treatment with control AS-Odn (n = 18). As VEGF is known to be an important factor for endothelial proliferation [23] and its synthesis is enhanced by hypoxia [23–25], we also measured the intracellular VEGF concentration as well as VEGF secretion in the supernatant in dependence of SHP-1 expression. Knock-down of SHP-1 resulted in a significant increase in the amount of intracellular (p<0.05; n = 10, Fig. 2B) and secreted (p<0.01; n = 8, Fig. 2C) VEGF during 24h hypoxia compared to control AS-Odn treatment. Since HIF-1α is known to be an important mediator of hypoxia driven cellular responses and an important transcription factor for VEGF under these conditions [8] we next asked whether SHP-1 may regulate cell growth and VEGF production via the HIF-1 pathway. Treatment with echinomycin, a pharmacological inhibitor of HIF-1, during hypoxia completely abolished the increase in cell growth resulting from SHP-1 knock-down during 24h hypoxia (p<0.05; n = 11, Fig. 2D) in comparison to cells treated with control AS-Odn. Furthermore, the elevation of intracellular VEGF concentration (p<0.05; n = 10, Fig. 2B) as well as amount of secreted VEGF (p<0.05; n = 8, Fig. 2C) by SHP-1 knock-down was also prevented by echinomycin. Finally, this effect was confirmed when using another pharmacological inhibitor of HIF-1α, apigenin (p<0.05; n = 10, Fig. 2B and C).

### SHP-1 regulates HIF-1α protein levels during hypoxia in HMEC

**Fig 3:**
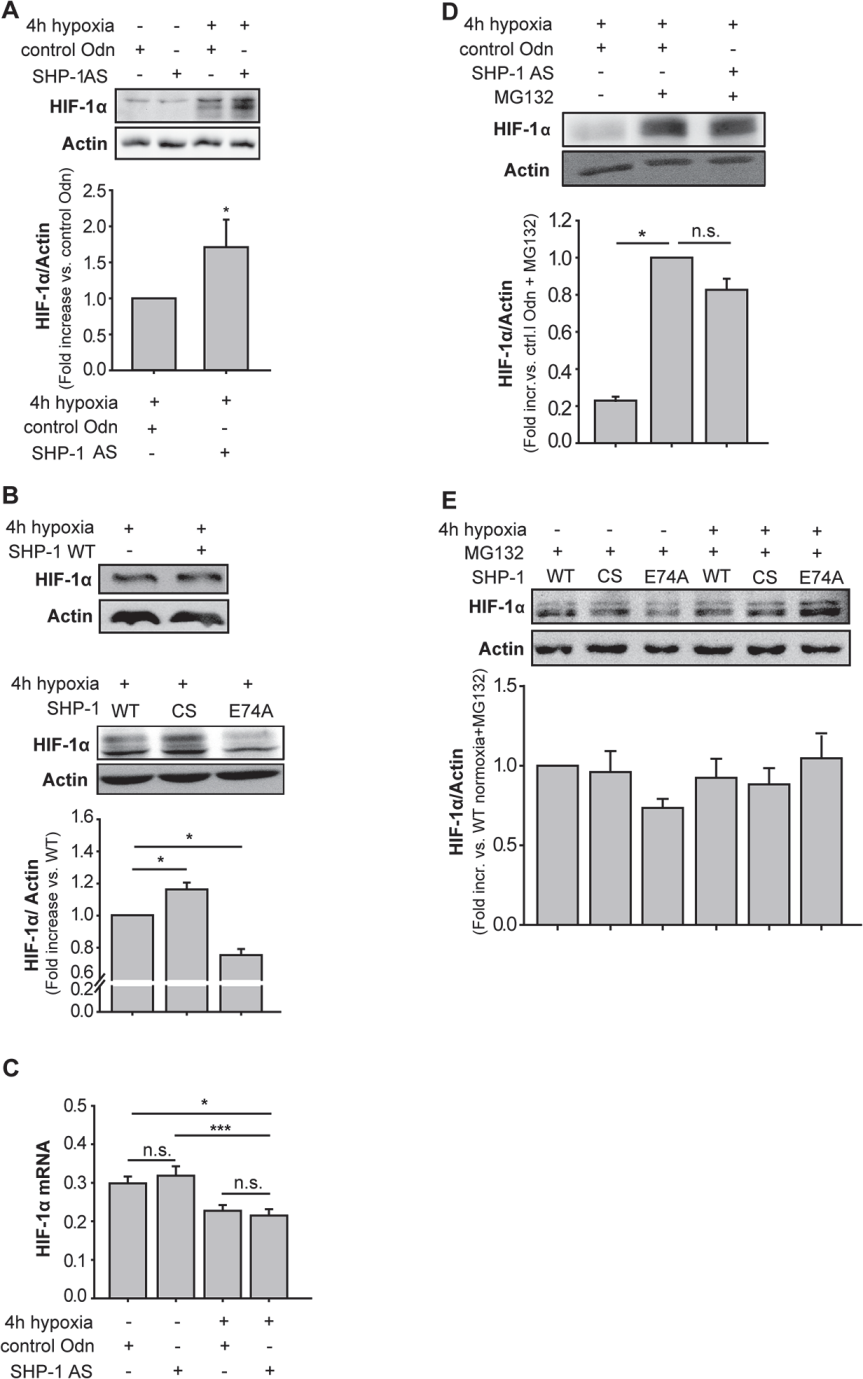
SHP-1 destabilizes HIF-1α protein during hypoxia. (A) HIF-1α protein levels were further enhanced by SHP-1 knock-down during hypoxia (4h) (*p<0.05, n = 4, lane 4) compared to control conditions (lane 3). Graph shows the protein band density of HIF-1α in relation to loading control β-Actin. (B) HIF-1α levels in non transfected cells was compared to SHP-1 WT expressing cells showing no influence of transfection on this (upper blot; n = 4). HMECs expressing inactive SHP-1 (CS) showed increased (*p<0.05; n = 8, lane 2 in lower blot), whereas expression of constitutively active SHP-1 (E74A) showed decreased (*p<0.05; n = 9, lane 3 in lower blot) levels of HIF-1α compared to wildtype (WT) SHP-1 (lane 1 in lower blot). Graph shows HIF-1α protein band density in relation to the loading control β-Actin. (C) HIF-1α mRNA was quantified by qRT-PCR. Hypoxia (4h) significantly decreased HIF-1α mRNA compared to normoxic conditions (*p<0.05; n = 10). However, SHP-1 knock-down had no effect on HIF-1α mRNA levels (n = 10). (D) The regulatory effect of SHP-1 on HIF-1α levels shown in (A) could not be observed when protein degradation was prevented by using the proteasome inhibitor MG132 (10μM) (n = 4). Graph shows HIF-1α protein band density in relation to the loading control β-Actin. (E) Differences in HIF-1α translation between cells expressing catalytic inactive (CS) and wildtype (WT) SHP-1 were not detected (n = 8) and the decreased HIF-1α accumulation seen in cells expressing constitutively active (E74A) SHP-1 was rescued upon inhibition of proteasomal inhibition (MG132 10μM) (n = 8).

As cell growth enhancement and increased VEGF synthesis by SHP-1 knock-down could be prevented by echinomycin, we next analyzed the effect of SHP-1 on HIF-1α protein levels. In cells treated with control AS-Odn hypoxia increased HIF-1α protein levels (n = 4, Fig. 3A), as expected. Interestingly, the induction of HIF-1α by hypoxia was even further enhanced by SHP-1 knock-down (p<0.05; n = 4, Fig. 3A). To confirm these data, HMECs were transfected with pDNA expressing either wildtype (WT), catalytically inactive (CS) or constitutively active (E74A) SHP-1. Compared to cells expressing the SHP-1 WT construct, overexpression of the inactive SHP-1 mutant (CS) resulted in increased HIF-1α protein levels (p<0.05; n = 8, Fig. 3B), whereas transfection with the constitutively active (E74A) SHP-1 construct led to decreased HIF-1α levels (p<0.05; n = 9, Fig. 3B). Having observed an influence of SHP-1 activity on HIF-1α protein levels, we next investigated whether SHP-1 influences the synthesis or the degradation of HIF-1α. Consequently, we assessed the transcription of HIF-1α by qRT-PCR. As shown in Fig. 3C hypoxia itself decreased the amount of HIF-1α mRNA compared to normoxic controls (p<0.05; n = 10), whereas SHP-1 knock-down had no additional effect in any of the two conditions (n = 10). Next, we analyzed translation and degradation of HIF-1α. As expected, treatment of HMEC with MG132 to block proteasomal degradation of HIF-1α further increased the amount of HIF-1α in hypoxic HMEC under control conditions (p<0.05; n = 4, Fig. 3D). However, the additional increase in HIF-1α protein level previously observed after SHP-1 knock-down could no longer be detected upon treatment with MG132 (n = 4, Fig. 3D). Equivalent to this, after MG132 treatment of cells expressing SHP-1 CS HIF-1α levels were returned to WT levels (n = 8, Fig. 3E). In accordance to this, the lost HIF-1α accumulation in cells expressing E74A was rescued back to WT levels when inhibiting proteasomal degradation during hypoxia (n = 8, Fig. 3E).

### SHP-1 regulates reactive oxygen species (ROS) formation in HMEC during hypoxia

**Fig 4:**
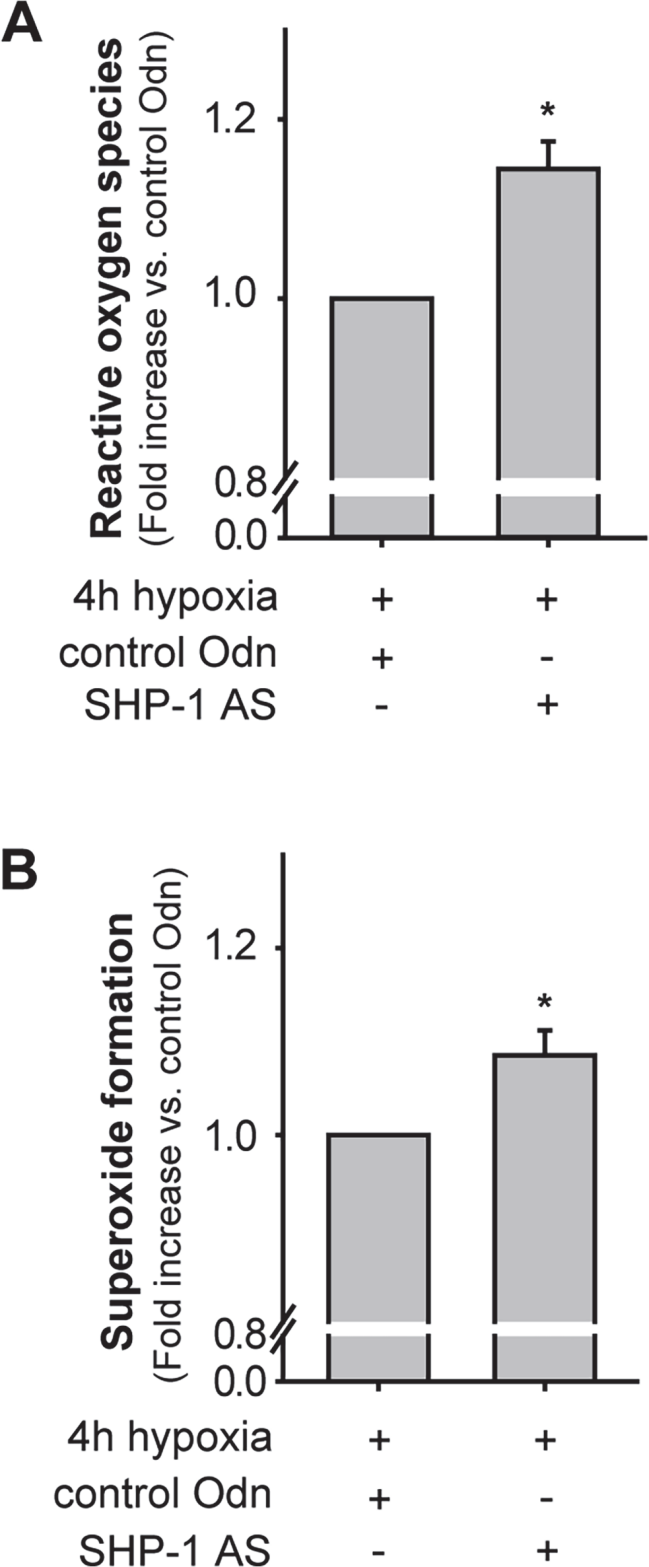
SHP-1 knock-down enhances ROS production during hypoxia. (A) SHP-1 knock-down enhanced ROS formation during hypoxia (*p<0.001; n = 12) compared to control AS-Odn treated cells. (B) SHP-1 knock-down enhanced hypoxia induced intracellular superoxide production (*p<0.05, n = 8), as assessed by DHE fluorescence.

As ROS have been shown to be essential for HIF-1α stability by inhibiting the activity of prolyl hydroxylases [11, 26, 27] and as we showed in a previous study that SHP-1 negatively regulates ROS production in non-stimulated and VEGF-treated EC [2], we hypothesized that SHP-1 may regulate the accumulation of HIF-1α protein by reducing ROS levels. Hence, we tested whether SHP-1 also effects endothelial ROS formation during hypoxia detecting the oxidation of H_2_DCF. SHP-1 silencing resulted in an increase in ROS formation (+ 15%) during hypoxia (p<0.001; n = 12, Fig. 4A) compared to control treatment. As we previously showed that SHP-1 regulates VEGF dependent superoxide production, we next investigated if SHP-1 has an impact on superoxide production during hypoxia by detecting dihydroethidium (DHE) fluorescence. As shown in Fig. 4B, SHP-1 knock-down also increased superoxide levels during hypoxia compared to treatment with control AS (p<0.05, n = 8).

## Discussion

The nonreceptor-type protein tyrosine phosphatase SHP-1 has been recognized as potential target to treat ischemic diseases [14–17, 28, 29]. Regulatory processes in which SHP-1 is involved during hypoxia are not fully understood. This study adds new aspects to the role of SHP-1 in endothelial hypoxic signaling and further underlines its potential as therapeutic target in ischemic diseases. This is the first study to show that SHP-1 is not only involved in VEGF receptor signaling, but that it also plays a crucial role in VEGF synthesis and secretion during hypoxia by regulating the transcription factor HIF-1.

In vivo studies of other groups showed an increased expression of SHP-1 upon ischemia in myocardial [15] and muscle cells [14], as well as in astrocytes [30]. An ex-vivo study working with retinal explants did not see any changes in SHP-1 expression or activity upon hypoxia [31]. In this study, we could only detect a moderate influence of 4h hypoxia on SHP-1 expression in HMEC. The stronger induction of SHP-1 upon hypoxia observed by others might be cell type specific or might only occur after longer exposure to hypoxia and not in acute hypoxia (4h) as used here. However, acute hypoxia is of great interest as it reflects conditions occurring in acute arterial occlusion like myocardial infarction. Consistent with the fact that SHP-1 and HIF-1α contain a nuclear localization signal (NLS) [32, 33] our data show that both proteins are mainly located in the nucleus of the endothelial cells with moderate expression in the cytoplasm. SHP-1 protein could only be detected after concentration by immunoprecipitation. This is probably due to lower SHP-1 expression levels in endothelial cells. Indeed, SHP-1 has been described to mainly be expressed in hematopoietic cells and thus show lower expression in endothelial cells. Tyrosine phosphorylation of HIF-1α could be detected but did not change between normoxic and hypoxic conditions. As HIF-1α is degraded during normoxia, the tyrosine phosphorylation may not be important under these conditions. Under hypoxic conditions however, tyrosine phosphorylated HIF-1α may play a role, as HIF-1α accumulates and may so constitute a binding site for SH2-containing proteins, such as SHP-1. Interestingly, we could also detect HIF-1α in precipitates of SHP-1. Thus, both proteins may even be part of the same protein complex

Upon SHP-1 silencing we observed increased endothelial cell growth under hypoxic conditions, which is in line with earlier studies [13, 14]. These earlier studies identified increased VEGFR-2 signaling [14] and increased expression of pro-angiogenic and anti-apoptotic factors [13] by SHP-1 knock-down, as being responsible for the observed effects highlighting the importance of SHP-1 as negative regulator of different pathways e.g. VEGF signaling. Here, we identified another aspect of how SHP-1 regulates endothelial cell growth during hypoxic conditions that has not been observed before. We found that SHP-1 inhibits synthesis of endothelial VEGF by accelerating HIF-1α protein degradation under hypoxic conditions. Our data show that SHP-1 knock-down increases intracellular and secreted VEGF. This is particularly interesting as endothelial VEGF is known to be crucial for hypoxia induced EC growth in terms of an autocrine loop mechanism [6, 12]. Furthermore, hypoxia has been shown to enhance the expression of the VEGF receptors [34, 35], which also contributes to the increased proliferative response during this condition. Cell growth and VEGF synthesis during hypoxia were impaired by echinomycin and apigenin, both pharmacological inhibitors of HIF-1α activity [36], indicating that both increased cell growth and VEGF synthesis are, at least partially, HIF-1α mediated. Interestingly, inhibition of HIF-1α by echinomycin completely blocked VEGF synthesis and secretion, whereas apigenin was shown to have a greater effect on VEGF synthesis and to a lesser extent, although still significant, on VEGF secretion. The same phenomenon can be seen in other studies using apigenin [37, 38], an effect which may be concentration dependent, as seen in the study of Mirzoeva et al. [38]. Nevertheless, the increase in hypoxic cell growth, VEGF synthesis and secretion by SHP-1 knock-down could be prevented by HIF-1α inhibition using echinomycin and apigenin, suggesting that SHP-1 is part of these HIF-1α dependent processes.

Analyzing the role of SHP-1 on HIF-1α protein levels we found that SHP-1 knock-down indeed further enhanced the induction of HIF-1α by hypoxia. The same results were obtained transfecting HMEC with an inactive SHP-1 construct. These findings were in accordance with the fact that expression of a construct containing a constitutively active SHP-1 decreased HIF-1α levels compared to expression of the wildtype construct. This experiment further confirms that the catalytic activity of SHP-1 is responsible for the observed effect and not merely expression of the protein. To the best of our knowledge this is the first time an explicit role of SHP-1 on HIF-1α induction in hypoxia and in endothelial cells is described. When investigating whether SHP-1 influences synthesis or degradation of HIF-1α we found that neither in normoxia nor in hypoxia SHP-1 knock-down seemed to influence HIF-1α transcription. However, upon inhibition of the HIF-1α degradation pathway, to investigate effects on translation as well as degradation, a difference in HIF-1α levels between SHP-1 knock-down cells and control cells or SHP-1 WT and SHP-1 CS expressing cells respectively could no longer be detected. This demonstrates that SHP-1 does not influence the translation of HIF-1α protein. Furthermore, as the decrease in HIF-1α levels in cells expressing constitutively active SHP-1 could be rescued by MG132 treatment, SHP-1 rather influences the degradation of HIF-1α. Regarding the mechanism how SHP-1 influences HIF-1α protein levels we know from previous studies that reactive oxygen species (ROS) stabilize HIF-1α under hypoxic [11, 26, 27] and normoxic [39–42] conditions. Previous data from our group showed that SHP-1 is a negative regulator of endothelial ROS formation under normoxic conditions [2]. In this study we could show that SHP-1 silencing also increases intracellular ROS formation, particularly superoxide molecules, during hypoxia. Hence, it is possible and plausible that the negative regulatory effect of SHP-1 on HIF-1α is the result of SHP-1 inhibiting endothelial ROS formation. The exact mechanism behind this regulation still needs to be elucidated and is part of our further research.

We conclude that SHP-1 promotes HIF-1α degradation under hypoxic conditions leading to a reduction in VEGF synthesis and secretion and thus impaired EC proliferation. A negative regulation of ROS by SHP-1 may have a potential mediator role in this signaling cascade. SHP-1 seems to prevent cellular overstimulation under hypoxic conditions and, hence, may be of importance in proliferative diseases e.g. in tumor angiogenesis. Finally, as inhibition of SHP-1 activity under hypoxia seems to be proangiogenic, SHP-1 may indeed constitute an interesting therapeutic target for ischemic diseases.
